# The association between the interval from biopsy to radical prostatectomy and biochemical recurrence in patients with intermediate- and high-risk prostate cancer

**DOI:** 10.3389/fonc.2024.1533800

**Published:** 2025-02-04

**Authors:** Carolin Siech, Mike Wenzel, Georgina Knoblich, Cristina Cano Garcia, Clara Humke, Felix Preisser, Miriam Traumann, Luis A. Kluth, Felix K. H. Chun, Philipp Mandel

**Affiliations:** ^1^ Goethe University Frankfurt, University Hospital, Department of Urology, Frankfurt am Main, Germany; ^2^ Martini-Klinik Prostate Cancer Center, University Hospital Hamburg-Eppendorf, Hamburg, Germany

**Keywords:** BCR, prostate cancer, prostate biopsy, radical prostatectomy, time to event

## Abstract

**Objective:**

To investigate the association between the interval from biopsy to radical prostatectomy (RP) and biochemical recurrence (BCR) in prostate cancer patients.

**Methods:**

Within a tertiary-care database (01/2014 to 06/2023), D’Amico intermediate- and high-risk prostate cancer patients were stratified according to interval from biopsy to RP (≤3 vs. >3-≤6 months). Kaplan-Meier survival analyses and Cox regression models addressed BCR.

**Results:**

Of 680 patients, 328 vs. 153 exhibited intermediate-risk prostate cancer and had interval from biopsy to RP ≤3 vs. >3-≤6 months. Similarly, 158 vs. 41 exhibited high-risk prostate cancer and had interval from biopsy to RP ≤3 vs. >3-≤6 months. Median interval from biopsy to RP was 59 vs. 113 days in intermediate- and 55 vs. 117 days in high-risk patients, respectively. In both intermediate- and high-risk patients, rates of adverse histopathological outcomes, namely pT3/pT4, pN1, and R1 status, did not differ according to interval from biopsy to RP. In survival analyses, three-year BCR-free survival rates were 82 vs. 88% in intermediate-risk (p=0.5) and 76 vs. 75% in high-risk patients (p=1). In multivariable Cox regression models, BCR did not significantly differ according to interval from biopsy to RP in intermediate- (hazard ratio 0.85, 95% confidence interval 0.49-1.46; p=0.5) and high-risk patients (hazard ratio 1.05, 95% confidence interval 0.50-2.22; p=0.9).

**Conclusions:**

Both intermediate- and high-risk prostate cancer patients with an interval from biopsy to RP >3-≤6 months did not differ from those treated with RP ≤3 months after biopsy, regarding adverse histopathological outcomes and BCR rates. Therefore, it might be safe to postpone RP up to six months.

## 1 Introduction

Radical prostatectomy (RP) represents a well-established curative treatment option in patients with non-metastatic prostate cancer (1, 2). Various factors can cause patients to postpone RP rather than undergo immediate surgery after being diagnosed with prostate cancer. These factors may include patient-related factors such as difficulty in decision making regarding curative treatment options due to the availability of alternative oncologically equivalent strategies as external beam radiotherapy (EBRT) (2). Some patients, especially those who are well-informed, ask for second or third opinions and need time to make their decision. Other patients may need further treatment to optimise comorbidities prior to RP (3). Further potential reasons for delayed treatment include limited resources of the health care system. Especially during the COVID-19 pandemic, but also afterward, surgical capacities have been limited due to staff shortages (4). These factors may lead to long waiting lists for elective urooncologic procedures, such as RP (4). The question for both, surgeons and patients, remains how long RP can be postponed safely.

In a preliminary study, we observed no differences between patients undergoing RP ≤3 months vs. >3 and ≤6 months after diagnosis for postoperative tumor characteristics, such as non-organ confined pathologic tumor stage, lymph node invasion, and positive surgical margins in patients with intermediate- and high-risk prostate cancer (5). Conversely, a Canadian multicenter study observed a higher risk of biochemical recurrence (BCR) following surgery in high-risk prostate cancer patients with time to RP ≥3 months (6), despite no differences in pathological outcomes (7).

We addressed this uncertainty and hypothesized that prostate cancer patients with an interval from biopsy to RP >3 and ≤6 months do not differ from those with an interval from biopsy to RP ≤3 months regarding histopathological outcomes at RP as well as BCR rates after RP. To address this hypothesis, we used a contemporary cohort of D’Amico intermediate- and high-risk prostate cancer patients treated with RP in a tertiary care referral center.

## 2 Materials and methods

### 2.1 Study population

**Figure 1:**
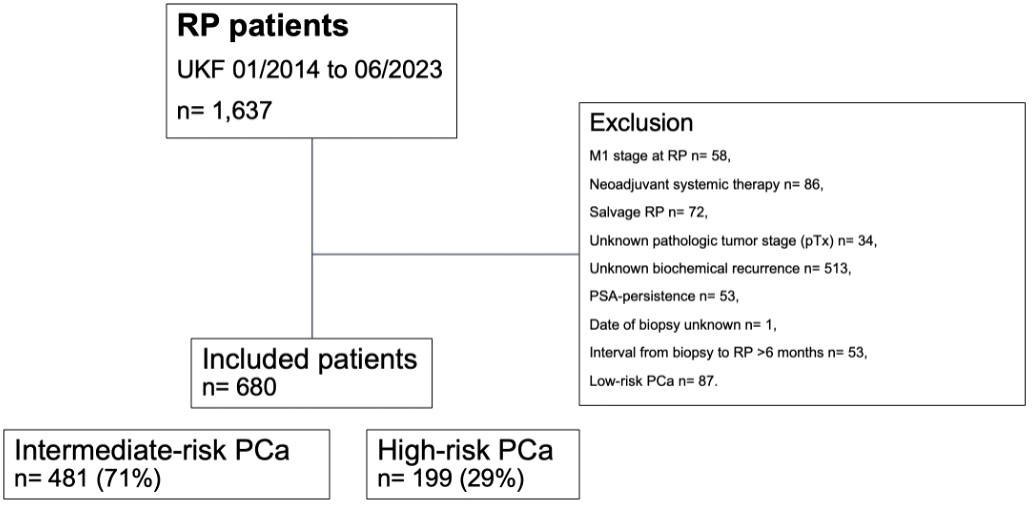
Consort diagram. PCa, prostate cancer; PSA, prostate-specific antigen; pT, pathologic tumor stage at surgery; RP, radical prostatectomy; UKF, University hospital Frankfurt.

Relying on a prospectively maintained database of a tertiary-care referral center, we retrospectively identified D’Amico intermediate- and high-risk histologically confirmed prostate cancer patients who were treated with open retropubic or robotic-assisted RP between January 2014 and June 2023 (**Figure 1**). Starting in November 2017, RP was routinely performed using the intraoperative frozen section technique (NEUROSAFE) and preserving the full functional length of the prostatic urethra (FFLU), as previously described by Preisser et al. (8).

Patients with D’Amico low-risk prostate cancer were not included in the study cohort, as they should be subjected to active surveillance in accordance with current guideline treatment recommendations (9–11). Inclusion criteria consisted of known follow-up regarding BCR and absence of prostate-specific antigen (PSA) persistence, defined as post-RP PSA of >0.1 ng/ml within six weeks after surgery (9–11). All patients with clinical suspicion of metastases at time of surgery (cM1), treatment with neoadjuvant systemic therapy (chemotherapy and/or hormonal therapy), previous radiation therapy of the prostate (salvage RP), unknown pathologic tumor stage (pTx), and unknown date of prostate biopsy were excluded. Due to limited sample size (n=30 for intermediate- and n=3 for high-risk prostate cancer), all patients with an interval from biopsy to RP >6 months were excluded from the study cohort.

Informed written consent to participate in this study was given by all patients. Prior to data collection, approval by the local ethics committee has been obtained. Reporting follows the precepts established by the Helsinki Declaration.

### 2.2 Definition of variables for analyses

All included D’Amico intermediate- and high-risk prostate cancer patients were stratified according to interval from biopsy to RP ≤3 months (≤90 days) vs. >3 and ≤6 months (>90 and ≤180 days). BCR represented the primary endpoint of the study and was defined according to the European Association of Urology (EAU) guidelines valid at the timepoint of BCR and the American Urological Association (AUA) guidelines as an initial serum PSA-value of ≥0.2 ng/ml, with a second confirmatory level of >0.2 ng/ml derived from patients’ self-reports in follow-up after RP (9–11). Upstaging was defined as non-organ confined stage (pT3/pT4 and/or pN1) in RP specimen in patients with clinically organ-confined stage (cT1 or cT2). Upgrading was defined as an increase of one or more Gleason Grade group from biopsy to RP specimen (12).

### 2.3 Statistical analyses

Four analytical steps were completed. First, clinical characteristics as well as histopathological outcomes, rates of nerve sparing surgery, and adjuvant radiation therapy were tabulated according to interval from biopsy to RP (≤3 vs. >3-≤6 months). For continuously coded variables, medians and interquartile ranges (IQR) were reported and for categorical variables, frequencies and respective proportions were recorded. Wilcoxon rank sum test assessed the statistical significance of medians’ differences for continuously coded variables and Pearson’s Chi-squared test examined the statistical significance in proportions’ differences for categorical variables. Moreover, Fisher’s exact test was used to compute an exact p-value when expected counts were less than ten. Second, estimated annual percentage changes (EAPC) for the proportion of patients treated with RP ≤3 months were tested with the least squares linear regression. Third, Kaplan-Meier plots depicted BCR-free survival rates after stratification according to interval from biopsy to RP. Finally, univariable and multivariable Cox regression models addressed BCR according to interval from biopsy to RP. Adjustment variables represented PSA-value at initial diagnosis (continuously coded), adverse histopathological outcomes at RP, namely pathologic tumor stage (pTstage), Gleason Grade group in specimen (ISUP grade), pathologic lymph node stage (pNstage), and positive surgical margin, as well as adjuvant radiation therapy. All analyses were separately performed in D’Amico intermediate- and high-risk prostate cancer patients.

All statistical tests were two sided, with a level of significance set at p<0.05. R software environment was used for statistical computing and graphics (R version 4.3.2; R Foundation for Statistical Computing, Vienna, Austria) (13).

## 3 Results

### 3.1 Clinical characteristics

Relying on our institutional tertiary-care database of 1,637 prostate cancer patients treated with RP between 01/2014 and 06/2023, 680 (42%) patients met the above-described inclusion criteria (**Figure 1**). Of these, 481 (71%) harbored intermediate-risk and 199 (29%) harbored high-risk prostate cancer (**Table 1**). Among intermediate-risk prostate cancer patients, the interval from biopsy to RP ranged from 16 to 177 days. Specifically, 328 (68%) patients had an interval from biopsy to RP ≤3 months and 153 (32%) patients >3 and ≤6 months. Median interval from biopsy to RP was 59 (IQR 44-72) vs. 113 (IQR 99-133) days, respectively. Over time, the proportion of patients treated with RP ≤3 months per year ranged from 88% in 2014 to 6% in 2023 (EAPC: −6.7%; 95% confidence interval [CI] −12.5 to −1.1). Among high-risk prostate cancer patients, the interval from biopsy to RP ranged from 14 to 180 days. Specifically, 158 (79%) patients had an interval from biopsy to RP ≤3 months and 41 (21%) >3 and ≤6 months. Median interval from biopsy to RP was 55 (IQR 42-69) vs. 117 (IQR 98-127) days, respectively. Over the study period, the proportion of patients treated with RP ≤3 months per year decreased from 80% in 2014 to 50% in 2023 (EAPC: −3.9%; 95% CI −7.2 to −0.7). Further clinical characteristics of the study cohort are summarized in **Table 1**.

**Table 1:** Clinical characteristics of 481 D’Amico intermediate- and 199 high-risk prostate cancer patients treated with radical prostatectomy (RP) between 01/2014 and 06/2023.

		Intermediate-risk prostate cancer, n= 481			High-risk prostate cancer, n= 199		
Characteristic		≤3 months n = 328 (68%)^1^	>3 and ≤6 months n = 153 (32%)^1^	p-value^2^	≤3 months n = 158 (79%)^1^	>3 and ≤6 months n = 41 (21%)^1^	p-value^2^
Interval from biopsy to RP (in days)		59 (44, 72)	113 (99, 133)	**<0.001**	55 (42, 69)	117 (98, 127)	**<0.001**
Age at surgery (in years)		65 (60, 70)	67 (62, 71)	**0.03**	67 (63, 72)	68 (64, 72)	0.3
Prostate volume (in ml)		40 (30, 50)	40 (30, 54)	0.1	44 (32, 55)	37 (30, 70)	0.6
PSA (in ng/ml)		6.7 (5.0, 9.4)	7.3 (5.1, 10.2)	0.4	10.3 (6.3, 22.8)	12.5 (6.2, 31.3)	0.5
cTstage	cT1	193 (59%)	101 (66%)	0.1	59 (37%)	16 (39%)	0.9
	cT2	135 (41%)	52 (34%)		85 (54%)	22 (54%)	
	cT3/cT4	0 (%)	0 (%)		14 (9%)	3 (7%)	
Gleason Grade Group in biopsy	1	33 (10%)	17 (11%)	**0.03**	4 (3%)	3 (7%)	**0.048**
	2	202 (64%)	110 (72%)		13 (8%)	5 (12%)	
	3	93 (28%)	26 (17%)		14 (9%)	6 (15%)	
	4	0 (%)	0 (%)		72 (46%)	21 (51%)	
	5	0 (%)	0 (%)		55 (35%)	6 (15%)	

^1^Median (interquartile range); n (%); ^2^Wilcoxon rank sum test; Pearson’s Chi-square test; Fisher’s exact test.

cTstage, clinical tumor stage; PSA, prostate-specific antigen; RP, radical prostatectomy. Bold values represent values significant at a level of significance set at p<0.05.

### 3.2 Histopathological outcomes, rates of nerve sparing surgery, and adjuvant radiation therapy

In 481 intermediate-risk prostate cancer patients stratified according to interval from biopsy to RP (≤3 vs. >3 and ≤6 months), rates of non-organ confined tumor stage (pT3/pT4) were 40 vs. 35%, rates of high-risk Gleason Grade group in specimen (ISUP grade 4/5) were 7 vs. 5%, rates of lymph node invasion (pN1) were 2 vs. 2%, and rates of positive surgical margins (R1) were 28 vs. 22% (**Table 2**). Upstaging from clinically organ-confined (cT1 or cT2) to pathological non-organ confined stage (pT3/pT4 and/or pN1) was experienced by 40 vs. 35%. Comparing Gleason Grade group in biopsy with those in RP specimen, upgrading was evident in 20 vs. 22% and downgrading in 19 vs. 17%. Rates of nerve sparing surgery were 92 vs. 93% in intermediate-risk patients with an interval from biopsy to RP ≤3 months vs. >3 and ≤6 months. Adjuvant radiation therapy rates were 8 vs. 6%, respectively.

**Table 2:** Histopathological outcomes, proportion of nerve sparing surgery, and adjuvant radiation therapy of 481 D’Amico intermediate- and 199 high-risk prostate cancer patients treated with radical prostatectomy (RP) between 01/2014 and 06/2023.

		Intermediate-risk prostate cancer, n= 481			High-risk prostate cancer, n= 199		
Characteristic		≤3 months n = 328 (68%)^1^	>3 and ≤6 months n = 153 (32%)^1^	p-value*^2^*	≤3 months n = 158 (79%)^1^	>3 and ≤6 months n = 41 (21%)^1^	p-value*^2^*
**pTstage**	pT2	198 (60%)	100 (65%)	0.3	46 (29%)	16 (39%)	0.2
	pT3/pT4	130 (40%)	53 (35%)		112 (71%)	25 (61%)	
**pNstage**	pN0	302 (92%)	141 (92%)	1.0	127 (80%)	37 (90%)	0.2
	pN1	8 (2%)	3 (2%)		28 (18%)	3 (8%)	
	pNx	18 (6%)	9 (6%)		3 (2%)	1 (2%)	
**Gleason Grade Group in specimen**	1/2/3	305 (93%)	145 (95%)	0.5	84 (53%)	27 (66%)	0.1
	4/5	23 (7%)	8 (5%)		74 (47%)	14 (34%)	
**Surgical margin status**	R0	230 (70%)	114 (75%)	0.3	81 (51%)	24 (59%)	0.7
	R1	91 (28%)	34 (22%)		73 (46%)	16 (39%)	
	Rx	7 (2%)	5 (3%)		4 (3%)	1 (2%)	
**Robotic-assisted radical prostatectomy**		275 (84%)	126 (82%)	0.7	106 (67%)	28 (68%)	0.9
**Nervesparing**		303 (92%)	143 (93%)	0.7	128 (81%)	35 (85%)	0.8
**Adjuvant radiation therapy**		26 (8%)	9 (6%)	0.4	31 (20%)	8 (20%)	1.0

^1^Median (interquartile range); n (%); ^2^Wilcoxon rank sum test; Pearson’s Chi-square test; Fisher’s exact test. Bold values represent values significant at a level of significance set at p<0.05.

In 199 high-risk prostate cancer patients stratified according to interval from biopsy to RP (≤3 vs. >3 and ≤6 months), rates of non-organ confined tumor stage (pT3/pT4) were 71 vs. 61%, rates of high-risk Gleason Grade group in specimen (ISUP grade 4/5) were 47 vs. 34%, rates of lymph node invasion (pN1) were 18 vs. 8%, and rates of positive surgical margins (R1) were 46 vs. 39% (**Table 2**). Upstaging from clinically organ-confined (cT1 or cT2) to pathological non-organ confined stage (pT3/pT4 and/or pN1) was experienced by 63 vs. 54%. Comparing Gleason Grade group in biopsy with those in RP specimen, upgrading was evident in 15 vs. 24% and downgrading in 44 vs. 46%. Rates of nerve sparing surgery were 81 vs. 85% in high-risk patients with an interval from biopsy to RP ≤3 months vs. >3 and ≤6 months. Adjuvant radiation therapy rates were 20 vs. 20%, respectively.

### 3.3 Biochemical recurrence in D’Amico intermediate-risk prostate cancer patients

**Figure 2:**
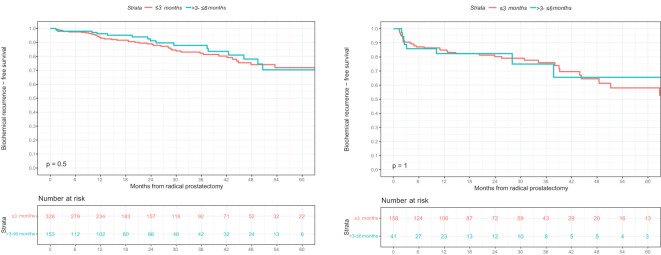
Kaplan-Meier survival analyses addressing biochemical recurrence (BCR)-free survival after radical prostatectomy (RP) according to interval from biopsy to RP in **(A)** D’Amico intermediate-risk and **(B)** high-risk prostate cancer patients. BCR, biochemical recurrence; BCRFS, biochemical recurrence-free survival; RP, radical prostatectomy.

Of all 481 intermediate-risk prostate cancer patients, BCR was experienced by 51 of 328 (16%) patients with an interval from biopsy to RP ≤3 months and by 18 of 153 (12%) patients with an interval from biopsy to RP >3 and ≤6 months. This was reflected in three-year BCR-free survival rates of 82% in patients with an interval from biopsy to RP ≤3 months and 88% in patients with an interval from biopsy to RP >3 and ≤6 months (p=0.5; **Figure 2A**). These rates resulted in a univariable hazard ratio (HR) for BCR of 0.84 (95% CI 0.49-1.45; p=0.5; **Table 3**). After multivariable adjustment for preoperative PSA-value, pTstage, Gleason Grade group in RP specimen, pNstage, surgical margin status, and adjuvant radiation therapy, the multivariable HR for BCR remained at 0.85 (95% CI 0.49-1.46; p=0.5; **Table 3**).

**Table 3:** Univariable and multivariable Cox regression models addressing rates of biochemical recurrence (BCR) after radical prostatectomy (RP), according to interval from biopsy to RP.

		Univariable			Multivariable*		
*D’amico risk group*	*Interval from biopsy to RP*	HR	95% CI	p-value	HR	95% CI	p-value
**Intermediate-risk prostate cancer**	**>3 and ≤ 6 months** (Ref. ≤3 months)	0.84	0.49, 1.45	0.5	0.85	0.49, 1.46	0.5
**High-risk prostate cancer**	**>3 and ≤ 6 months** (Ref. ≤3 months)	1.02	0.49, 2.09	1.0	1.05	0.50, 2.22	0.9

*adjusted for preoperative PSA-value, pTstage, Gleason Grade group in specimen, pNstage, surgical margin status, and adjuvant radiation therapy.

CI, confidence interval; HR, hazard ratio; RP, radical prostatectomy. Bold values represent values significant at a level of significance set at p<0.05.

### 3.4 Biochemical recurrence in D’Amico high-risk prostate cancer patients

Of all 199 high-risk patients, BCR was experienced by 41 of 158 (26%) patients with an interval from biopsy to RP ≤3 months and by 9 of 41 (22%) patients with an interval from biopsy to RP >3 and ≤6 months. This was reflected in three-year BCR-free survival rates of 76% in patients with an interval from biopsy to RP ≤3 months and 75% in patients with an interval from biopsy to RP >3 and ≤6 months (p=1; **Figure 2B**). These rates resulted in a univariable HR for BCR of 1.02 (95% CI 0.49-2.09; p=1; **Table 3**). After multivariable adjustment, the multivariable HR for BCR remained at 1.05 (95% CI 0.50-2.22; p=0.9; **Table 3**).

## 4 Discussion

Within the current study, we hypothesized that both D’Amico intermediate- as well as high-risk prostate cancer patients with interval from biopsy to RP ≤3 months compared to those with interval from biopsy to RP >3 and ≤6 months do not differ in BCR rates after RP. Relying on a contemporary cohort of RP-treated D’Amico intermediate- and high-risk prostate cancer patients at a tertiary care referral center between 01/2014 and 06/2023, we made several noteworthy observations.

First, among 481 D’Amico intermediate-risk prostate cancer patients, 328 (68%) patients had an interval from biopsy to RP ≤3 months and 153 (32%) patients had an interval from biopsy to RP >3 and ≤6 months. Similarly, among 199 high-risk prostate cancer patients, 158 (79%) patients had an interval from biopsy to RP ≤3 months and 41 (21%) patients had an interval from biopsy to RP >3 and ≤6 months. These distributions of intervals from biopsy to RP do not only validate the hypothesis that the majority of patients receives curative treatment within three months. Moreover, they are also consistent with the distributions of intervals from biopsy to RP reported by other prostate cancer centers in Europe (14) and North America (15–17).

Second, we identified no differences regarding rates of non-organ confined tumor stage (pT3/pT4), high-risk Gleason Grade group in RP specimen (ISUP grade 4/5), lymph node invasion (pN1), and positive surgical margins (R1). Moreover, no differences in the rates of upstaging from organ-confined to non-organ-confined stage were observed. Conversely, in high-risk but not in intermediate-risk prostate cancer patients, upgrading was more frequent in those with an interval from biopsy to RP of >3 and ≤6 months compared to those with an interval of ≤3 months. The higher upgrading rate in high-risk prostate cancer patients with an interval from biopsy to RP of >3 and ≤6 months (34 vs. 15%) may be attributed to the higher rate of Gleason Grade group 5 in biopsies in patients who underwent RP within ≤3 months (35 vs 15%). In consequence, the findings reported within the present study may suggest that a treatment delay of up to six months does not impair histopathologic outcomes at RP in both intermediate- as well as high-risk prostate cancer patients. Hereby, the current results confirm previous studies in which interval from biopsy to RP represented the variable of interest (5, 16, 18–22).

Third, the proportion of patients who received adjuvant radiation therapy was 8 vs. 6% in intermediate-risk (p=0.4) and 20 vs. 20% in high-risk prostate cancer patients who were treated with RP ≤3 vs. >3 and ≤6 months (p=1), respectively. The above findings demonstrate that not only histopathological outcomes at RP but also the rates of further treatments do not differ significantly between prostate cancer patients treated at ≤3 vs. >3 and ≤6 months after diagnosis.

Fourth, we observed no differences in BCR rates between prostate cancer patients who underwent RP ≤3 vs. >3 and ≤6 months after biopsy. Specifically, three-year BCR-free survival rates were 82 vs. 88% in patients with intermediate-risk prostate cancer (p=0.5) and 76 vs. 75% in patients with high-risk prostate cancer treated with RP ≤3 vs. >3 and ≤6 months after biopsy (p=1). Moreover, after multivariable adjustment for preoperative PSA-value, pTstage, Gleason Grade group in RP specimen, pNstage, surgical margin status, and adjuvant radiation therapy, interval from biopsy to RP did not receive independent predictor status for BCR after RP in both intermediate- and high-risk prostate cancer patients (p=0.5 and p=0.9). In intermediate- and high-risk prostate cancer patients treated with RP, a contemporary metanalysis by Laukhtina et al. included five studies which did not identify any significant association between treatment delay and BCR (23). Conversely, four included studies reported an unfavorable impact of treatment delay on BCR (23). However, the definitions of RP delay varied significantly between the included studies, ranging from continuously coded interval from biopsy to RP (6, 24) to cutoffs at 4-6 weeks (18–20) to >12 months (25). These differences render direct comparisons of such studies impossible.

Taken together, we identified no differences between immediate and delayed RP in either the intermediate- or high-risk groups regarding histopathological characteristics at RP, the proportion of patients receiving adjuvant radiation therapy, as well as BCR rates in patients treated with RP within six months. The observations recorded within the present study suggest that patients with intermediate- and high-risk prostate cancer may be reassured about waiting to pursue RP for up to 6 months after biopsy. Therefore, the above findings are of high clinical value in patient counselling and treatment decision making in times of limited surgical capacities and staff shortages.

Besides its strengths, the current study has limitations. First, due to its retrospective nature, a potential for residual selection biases between patients who underwent immediate compared to those who underwent delayed surgery remained, despite systematic adjustment for biases and confounders in multivariable models. Especially patients with very high-risk features might be allocated to the “early treatment” group (≤3 months). This might be indicated by the higher Gleason Grade group in biopsies in the “early treatment” groups. This limitation is applicable to all previous studies relying on a retrospective study design (5–7, 14–16, 18–22, 24–26). However, it is highly unlikely that a prospective trial randomizing patients to immediate vs. delayed RP will ever be initiated and completed. Second, our single-institutional database is limited by sample size. Therefore, the association between interval from biopsy to RP >6 months and adverse histopathological outcomes, adjuvant radiation therapy rates, as well as BCR rates could not be addressed in the current study. Moreover, time to event analyses focusing specifically on patients with very high-risk prostate cancer were not possible. Third, postoperative follow-up within our study cohort was also limited. In consequence, other study endpoints that could be equally as interesting as BCR, namely metastasis, cancer-specific, other-cause, or overall mortality could not be addressed within the current database.

## 5 Conclusions

Both intermediate- and high-risk prostate cancer patients with an interval from biopsy to RP >3 and ≤6 months did not differ from those treated within 3 months after biopsy, regarding adverse pathologic outcomes and BCR rates after RP. Therefore, it might be safe to postpone RP up to 6 months.

## Data Availability

The raw data supporting the conclusions of this article will be made available by the authors, without undue reservation.
